# Evolutionary history shapes variation of wood density of tree species across the world

**DOI:** 10.1016/j.pld.2024.04.002

**Published:** 2024-04-10

**Authors:** Fangbing Li, Hong Qian, Jordi Sardans, Dzhamal Y. Amishev, Zixuan Wang, Changyue Zhang, Tonggui Wu, Xiaoniu Xu, Xiao Tao, Xingzhao Huang

**Affiliations:** a Anhui Province Key Laboratory of Forest Resources and Silviculture, Anhui Agricultural University, Hefei 230036, China; b State Key Laboratory of Subtropical Silviculture, Zhejiang A&F University, Hangzhou 311300, China; c Research and Collections Center, Illinois State Museum, 1011 East Ash Street, Springfield, IL 62703, USA; d CREAF, Cerdanyola del Vallès, Barcelona 08193, Spain; e Global Ecology Unit CREAF-CSIC-UAB, CSIC, Bellaterra, Barcelona 08193, Spain; f Key Laboratory of Ecosystem Carbon Source and Sink, China Meteorological Administration (ECSS-CMA), School of Applied Meteorology, Nanjing University of Information Science & Technology, 219 Ningliu Road, Nanjing 210044, China; g Department of Natural Resources Management, Lakehead University, Thunder Bay, Ontario P7B 5E1, Canada; h School of Forestry & Landscape of Architecture, Anhui Agricultural University, Hefei 230036, China; i Research Institute of Subtropical Forestry, Chinese Academy of Forestry, Hangzhou 311400, China

**Keywords:** Wood density, Phylogeny, Angiosperms, Gymnosperms, Climate factors, Biophysical parameters

## Abstract

The effect of evolutionary history on wood density variation may play an important role in shaping variation in wood density, but this has largely not been tested. Using a comprehensive global dataset including 27,297 measurements of wood density from 2621 tree species worldwide, we test the hypothesis that the legacy of evolutionary history plays an important role in driving the variation of wood density among tree species. We assessed phylogenetic signal in different taxonomic (e.g., angiosperms and gymnosperms) and ecological (e.g., tropical, temperate, and boreal) groups of tree species, explored the biogeographical and phylogenetic patterns of wood density, and quantified the relative importance of current environmental factors (e.g., climatic and soil variables) and evolutionary history (i.e., phylogenetic relatedness among species and lineages) in driving global wood density variation. We found that wood density displayed a significant phylogenetic signal. Wood density differed among different biomes and climatic zones, with higher mean values of wood density in relatively drier regions (highest in subtropical desert). Our study revealed that at a global scale, for angiosperms and gymnosperms combined, phylogeny and species (representing the variance explained by taxonomy and not direct explained by long-term evolution process) explained 84.3% and 7.7% of total wood density variation, respectively, whereas current environment explained 2.7% of total wood density variation when phylogeny and species were taken into account. When angiosperms and gymnosperms were considered separately, the three proportions of explained variation are, respectively, 84.2%, 7.5% and 6.7% for angiosperms, and 45.7%, 21.3% and 18.6% for gymnosperms. Our study shows that evolutionary history outpaced current environmental factors in shaping global variation in wood density.

## Highlights

• Wood density shows a significant phylogenetic signal.
• Phylogeny and species together explain the vast majority of total wood density variation at a global scale.
• Evolutionary history outpaces current environmental factors in explaining global variation in wood density.

## 1 Introduction

Wood density is a key functional trait of woody plants, particularly trees. It correlates with a range of morphological, mechanical, physiological, and ecological properties of trees (Chave et al., 2009; Hietz et al., 2013). Variation in wood density is closely associated with variation in rate of diameter growth, rate of mortality, timing of reproduction, hydraulic capacities of the stem, and the relative mechanical strength of a woody plant (Carlquist, 1975; Enquist et al., 1999). Denser wood has more carbon and energy content per unit volume, compared with lighter wood (Enquist et al., 1999). Mechanical support increases with tissue density, which can be linked to the maximum height of a woody plant for a given stem diameter. Indeed, the maximum height of a woody plant for a given stem diameter critically relies on the density of wood (Swenson and Enquist, 2007). The increased mechanical support provided by higher wood densities also increases resistance to stem breakage due to extrinsic forces (e.g., wind), which would reduce mortality and ultimately influence plant community structure (Putz et al., 1983; ter Steege and Hammond, 2001).

Wood density is an important parameter in models predicting wood quality and carbon stocks (weaker predictor than tree diameter but stronger predictor than tree height) (Bouriaud et al., 2004; Chave et al., 2005, 2009; Flores and Coomes, 2011; Kerfriden et al., 2021; Wang et al., 2023). Considering variation in wood density among species and across ecological gradients in biomass modeling can result in a better prediction of dynamic changes in forest woody biomass (Baker et al., 2004; Stegen et al., 2009), which is crucial for estimating carbon storage per unit area since *ca*. 90% of tree aboveground biomass is woody (Chave et al., 2009). Wood density also plays a central role in studies of global woody biomass dynamics, and its variation among species represents key linkages between tree growth strategies and ecosystem function across forests worldwide (Phillips et al., 2019; Maynard et al., 2022).

Previous studies have demonstrated the significance of environmental factors, such as climatic variables, soil characteristics, and stand structure, in shaping wood density, which is a key functional trait driven by evolutionary and ecological processes (Kurz-Besson et al., 2016; Sette Jr et al., 2016; Ibanez et al., 2017; Bytebier et al., 2022). However, what is the main drivers of wood density at a global scale remain unknown, with conflicting evidence for the primary role of temperature (Bouriaud et al., 2004; Thomas et al., 2004; Kimberley et al., 2017; Fajardo et al., 2022), versus precipitation (Stoehr et al., 2009; Zhang et al., 2011; Camarero et al., 2017). Wood density variation is also further related with multiple factors, such as soil organic carbon and nitrogen content, stand age and density (Baker et al., 2004; Muller-Landau, 2004; Chave et al., 2006; Oliveira et al., 2021), making large-scale spatial distribution patterns of wood density uncertain. At local scales, environmental filtering and spatial constraints are considered crucial determinants of wood density, while phylogeny has played a relatively minor role (Borg et al., 2013). However, the evolutionary constraints of wood density by biotic and abiotic forces in different evolutionary lineages, such as angiosperms versus gymnosperms and subgroups within them, are still unclear. Therefore, a global analysis of the spatial patterns and drivers of wood density is essential for a better understanding of forest carbon dynamics at a large scale.

In forest succession, fast-growing tree species tend to be better colonists and dominate the early stages of the succession, while slow-growing species dominate the later stages (Uhl and Jordan, 1984; Lugo and Scatena, 1996). This pattern is reflected in variation in wood density, where fast-growing pioneer species have lower wood densities, while late-succession species have higher wood densities (Köhler et al., 2000). Wood density and tree growth rate have a strong negative correlation (Ogle et al., 2014; Greenwood et al., 2017), and understanding the mechanisms driving the trade-off between low wood density and high growth rates in both angiosperm and gymnosperm trees (ter Steege and Hammond, 2001; Wright et al., 2003; Muller-Landau, 2004) requires further investigation of differences in wood density among different taxonomic and ecological groups of species. On the other hand, more stress-tolerant species, particularly to drought conditions, have higher wood density than species of more resource-rich environments (Cielo-Filho, 2021; Liang et al., 2021; Serra-Maluquer et al., 2022).

Functional ecology posits that the key functional traits that drive organismal performance are influenced by variations in survival and reproduction (i.e., fitness) across varying environments (McGill et al., 2006; Westoby and Wright, 2006; Huang et al., 2023b). Evolutionary inheritance, driven by common ancestry, is often used to explain adaptive changes in traits related to tree growth and life-history strategies such as wood density. Studies based on family-level phylogeny in seed plants suggest that more closely related species are more similar in wood density, compared to distantly related ones. Differences in wood density impact the morphology and function of the entire plant, leading to interspecific variation (Swenson and Enquist, 2007; Savva et al., 2010). Phylogeny is an important factor explaining the functional adaptation of wood density (Swenson and Enquist, 2007; Savva et al., 2010; Zhang et al., 2011; Ibanez et al., 2017; Wang et al., 2022); phylogenetic relatedness among species may help predict wood density of different species based on those species whose wood density is known. Moreover, other variables related with evolutionary process such as recent convergence or divergence in traits can also explain part of the variation of wood density when comparing different species, which has been observed in other plant traits such as foliar elemental composition (Sardans et al., 2021).

We compiled a large global dataset of 27,297 wood density samples from various forest regions across the world to explore biogeographical patterns and phylogenetic structure of wood density at a global scale and to carry out a comprehensive assessment of the relative roles of current environmental factors and phylogeny, including long-term evolution processes (assessed by phylogenetic tree) and other factors related to taxonomy such as recent convergent and divergent evolution, in explaining the global wood density variation. Our study aims to answer the following three questions: (1) What are the patterns of wood density variation across vegetation biomes and climate zones? (2) Are there phylogenetic signals in wood density for all tree species as a whole, for trees in angiosperms and gymnosperms separately, and for trees in different climate zones (e.g., tropical, temperate, and boreal)? (3) What is the relative importance of current environmental factors and evolutionary history in shaping global wood density variation? We hypothesize that wood density would vary among different biomes and different climatic zones and show a significant phylogenetic signal; furthermore, we hypothesize that the wood density variations across global biomes are mainly driven by evolutionary history and current environmental conditions, especially evolutionary history.

## 2 Materials and methods

### 2.1 Data compilation

The present study used a large global dataset of wood density, which was primarily compiled from two sources: Global Wood Density database (Zanne et al., 2009) and Global Plantation Forest Carbon database (Bukoski et al., 2022). Data in these two global databases were derived mainly from the plant taxonomy and wood technology literature and studies that quantified biomass stocks. To expand the dataset, we obtained data from the TRY plant trait database (https://www.try-db.org/TryWeb/dp.php) using the DataName “Wood density” (Kattge et al., 2020).

**Fig. 1:**
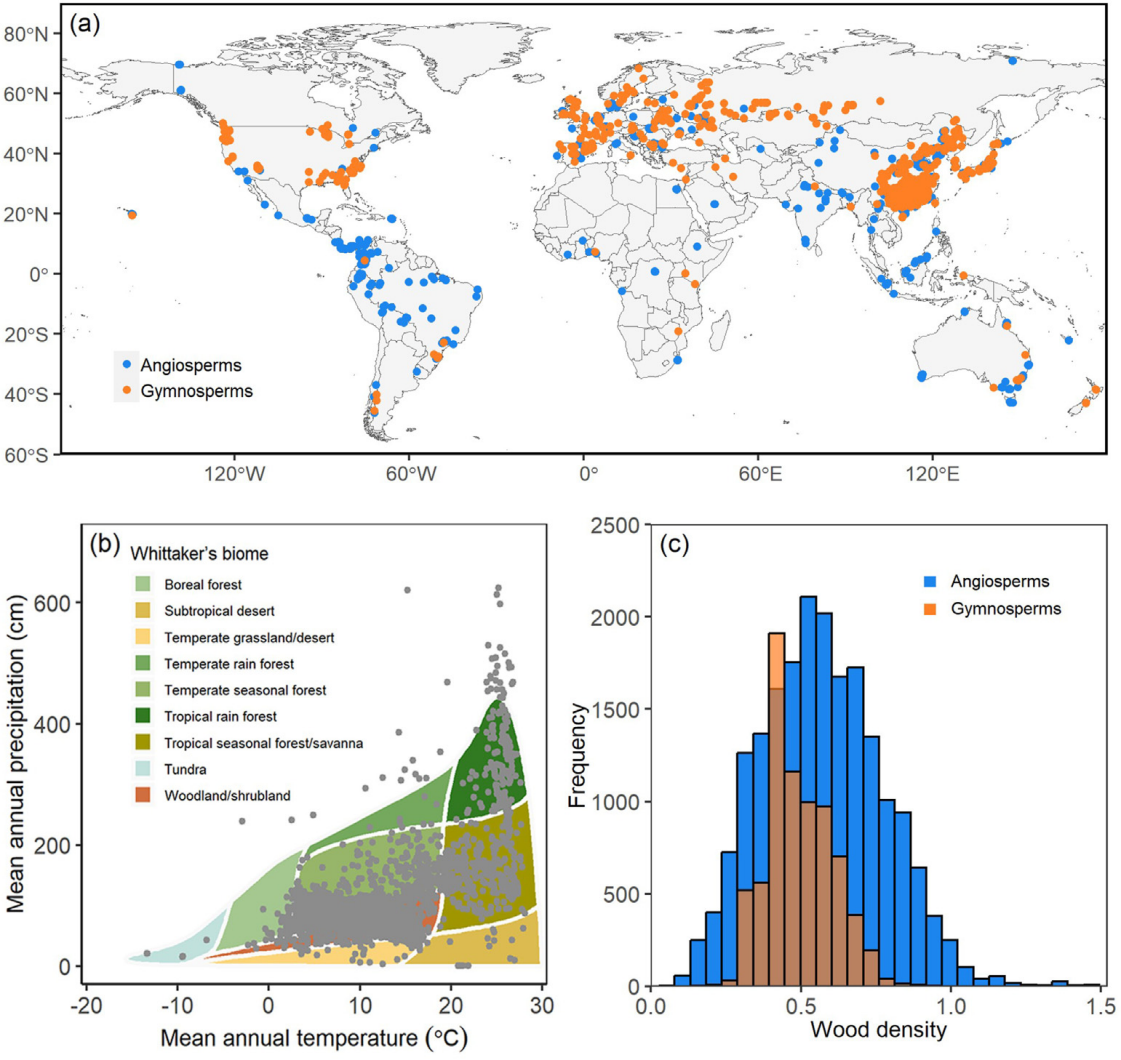
Global dataset of wood density in compiled data. (a) Geographical distribution of sites across the world. Blue circles represent sites of angiosperms; orange circles represent sits of gymnosperms. The map is created in R 4.2.2 (URL https://www.R-project.org/). (b) Distributions of the study sites across Whittaker's biomes. (c) Frequency distribution of 27,297 data points according to wood density of angiosperms and gymnosperms.

To obtain a final consistent and homogeneous dataset in terms of study's methodology, we reviewed each measurement of these databases to determine whether it was to be included in our study. Data points were excluded if they do not have specific latitude and longitude coordinates or they cannot be estimated based on available geographic information, or if they do not have complete scientific names at species or lower level. Botanical nomenclature was standardized according to World Plants (https://www.worldplants.de), using the package U.Taxonstand (Zhang and Qian, 2023). Tree species in our dataset were identified according to Beech et al. (2017), and non-tree species were excluded from the present study. As a result, our final dataset included 27,297 measurements (samples) of wood density from 2621 tree species in 953 genera and 156 families, located in 7555 distinct sites worldwide (Fig. 1a, 1b and Table S1).

Previous studies have rarely identified for certain which environmental factors are key drivers of wood density. However, with respect to climatic variables, mean annual temperature (MAT) and mean annual precipitation (MAP) are among the most important climatic variables influencing plant distribution, and have been used exclusively to represent climatic conditions in many botanical studies (e.g., Lu et al., 2018; Wu and Wiens, 2022). In particular, Whittaker's (1975) biome classification system was built based on these two climatic variables. Furthermore, these two climatic variables have been used in summarizing information on variation of wood density across climatic gradients (e.g., Swenson and Enquist, 2007). Accordingly, we used mean annual temperature and mean annual precipitation to represent climatic conditions in this study. We obtained data for MAT and MAP from the CHELSA database (http://chelsa-climate.org/), corresponding to bio1 and bio12, respectively. Normalized Difference Vegetation Index (NDVI) has been commonly used as a measure of primary productivity (Pettorelli et al., 2011; Vicente-Serrano et al., 2016; Adan et al., 2023); we included NDVI in this study. NDVI data were extracted from the Global GIMMS NDVI3g.v1 dataset (1981–2015) in the National Tibetan Plateau/Third Pole Environment Data Center (Tucker et al., 2005; Pinzon and Tucker, 2014). Soil clay content (termed ‘clay’ hereafter) and soil organic carbon concentration (SOC) are among the most important soil variables influencing tree growth and wood density (Kimmins, 2004). Thus, we used these soil variables in this study. Soil data were derived from the SoilGrids database22 (Hengl et al., 2017). In addition, the slope and aspect of a site can also affect tree growth rate, which can in turn influence wood density (Kimmins, 2004), we also included them as explanatory variables in our initial models. We used 30-m DEM data from the NASA Shuttle Radar Topographic Mission to determine the site slope (termed ‘slope’ hereafter) and site aspect (termed ‘aspect’ hereafter) using SAGA-GIS software v.2.1.4 (Conrad et al., 2015). These variables have been considered as drivers or correlates of tree growth and wood density (Baker et al., 2004; Muller-Landau, 2004; Chave et al., 2006; Onoda et al., 2010; Diaconu et al., 2016; Nabais et al., 2018; Oliveira et al., 2021).

We analyzed all tree species in the data together. In addition, we divided the tree species into two major taxonomic groups, i.e., gymnosperm and angiosperm, because it is broadly known that wood structure differs substantially between gymnosperm (‘softwood’) trees and angiosperm (‘hardwood’) trees (Hardin et al., 2001). Within angiosperms, in addition to analyzing angiosperms as a whole, we conducted analyses for six major monophyletic clades of angiosperms (Campanulids, Fabids, Lamiids, Magnoliids, Malvids and Monocots), which collectively accounted for 87% of all angiosperm tree species examined in this study. We were also interested in investigating the role of evolutionary legacy on driving wood density in the main climate types, and with this aim both gymnosperms and angiosperms were divided into three climate groups (tropical, temperate and boreal), based on the mean annual temperature of the study sites of each species. We considered a species being tropical if its average of mean annual temperature > 20 °C, temperate if its average of mean annual temperature being ≤ 20 °C and ≥ 5 °C, or boreal if its average of mean annual temperature being < 5 °C (Ricklefs, 2008). Each species was only assigned to one of the three climatic groups. Temperate region had the largest number of samples (n = 13,851), followed by tropical region (n = 12,259) and boreal region (n = 1187).

### 2.2 Data analysis

#### 2.2.1 Classification of biomes

To explore the biogeographical patterns of global wood density variation, we analyzed the variation of wood density across different biomes. Following the criteria of the classic Whittaker's biome classification system based on mean annual precipitation and mean annual temperature (Whittaker, 1975), the vast majority (98.15%) of our study sites were located in the nine biomes defined by Whittaker: tundra, boreal forest, temperate seasonal forest, temperate rain forest, tropical rain forest, tropical seasonal forest/savanna, subtropical desert, temperate grassland/desert and woodland/shrubland.

#### 2.2.2 Phylogenetic analysis of wood density

To assess the role of evolutionary history in shaping the variation of current wood density among species, we conducted phylogenetic analyses based on model parameters in the R environment (Ihaka and Gentleman, 1996; Pagel, 1999; Jin and Qian, 2023).

First, we used the functions build.nodes.1 and Scenario 3 in the package U.PhyloMaker (Jin and Qian, 2023) to generate a phylogenetic tree for the species present in our database to facilitate phylogenetic analysis (Molina-Venegas and Rodriguez, 2017). The structure of the phylogenetic tree demonstrates the historical evolutionary process of species diversity, because biological evolution events were recorded in the branches of the phylogenetic tree, and the common ancestor of all species is located at the root of a tree. Generations exist at the nodes of the branches from the root to the tips of branches, which are measured in time units or evolutionary units. When conducting an analysis for a specific species group, we extracted a sub-phylogenetic tree from the phylogenetic tree for the analysis.

Second, the phylogenetic signal of wood density was assessed using Pagel's λ statistics, which is the phylogenetic signal statistic commonly used to evaluate phylogenetic signal of a focal trait with respect to a phylogenetic tree (Pagel, 1999; Qian and Zhang, 2014). Pagel's λ has been shown to be a robust measure of phylogenetic signal ranging from 0 to 1 (Molina-Venegas and Rodriguez, 2017), where a value of 1 indicates that the evolution of the trait matches expectations under the Brownian motion model of evolution, while a value of 0 indicates a random distribution of a trait with respect to the phylogeny (Qian and Zhang, 2014).

We used the function *phylosig* implemented in the package phytools to compute the λ metric (Revell, 2012; Jin and Qian, 2023). The statistical significance of λ was tested by comparing the likelihood of the observed λ value with the likelihood of a model that assumes complete phylogenetic independence (Felsenstein, 1985; Pagel, 1999; Münkemüller et al., 2012). Phylogenetic signal was considered significant (*p* < 0.05) when the observed value was greater than the null model value derived from 1000 randomizations.

#### 2.2.3 The relative contribution of environmental factors and evolutionary history to global wood density variation

To explore how current environmental factors and evolutionary history have affected global wood density variation, we conducted analyses at the site-species level. We calculated the average wood density for every species within the same sampling site and used a Bayesian phylogenetic linear mixed model in the R package “*MCMCglmm*” (Hadfield, 2010) to disentangle the relative contributions of current environmental factors and evolutionary history to the global wood density variation. We used the factors of MAT, MAP, clay, SOC, slope, aspect and NDVI to fit all tree species as a whole, and angiosperms and gymnosperms separately, and the factors were filtered according to the fitting results. To prevent overfitting, we finally selected MAT, MAP, clay and NDVI as the most important factors for all the data and also for the different plant groups by comparing the AICs derived from the “*dredge*” function of the *MuMIn* package (Burnham and Anderson, 2002). We used the most important environmental factors identified above as fixed factors, with the phylogeny and species as random factors (Sardans et al., 2021). For the phylogeny, we used the phylogenetic tree constructed based on an available mega-phylogeny of vascular plants (Jin and Qian, 2023), as described above. The random factors described the effect of evolutionary history on wood density variation, with the phylogenetic term accounting for the variation in shared ancestry in terms of evolutionary time distance among species, and the species term accounting for the interspecific variation independent of the shared ancestry; this, for instance, considers other legacy variables such as recent divergent and convergent evolution. Effectively, for instance, two species separated long time ago in the phylogenetic tree can have some characters more similar than expected by the phylogenetic time separation between them, this can occur if they coincided in similar environment in the past times, and thus have more similarity in some variables between them than expected by phylogenetic separation (evolutionary convergence). On the other hand, the contrary situation is that nearby species in phylogenetic tree evolved in separate ways faster in recent times to be submitted to very different environmental situations, making its “studied variables” more distinct than the expected by phylogenetic distance between them (evolutionary divergence) (Sardans et al., 2021; Vallicrosa et al., 2022). This approach has been used in recent studies exploring phylogenetic effect on biological and ecological traits (e.g., Sardans et al., 2021).

Following Sardans et al. (2021), we quantified the relative importance of phylogeny, species, and environmental variables (MAT, MAP, clay and NDVI) using the random forest method. The results of this analysis include, but are not limited to, *p*-value from Monte Carlo sampling by Markov Chain for each environmental variable and the proportions of the variance explained by all the model (fixed + random) terms, by phylogeny, and by species. This analysis also reports the proportion of variation in wood density that is not explained by phylogeny and species but is explained by environmental variables. Because this analysis does not report the proportion of the variation in wood density that is explained by environmental variables in each model, we conducted multiple linear regressions to assess the amount of the variation in wood density that is explained by the environmental variables included in each model. We assessed the relative importance of phylogeny, species, and environmental variables on wood density by comparing the variation in wood density that was explained by each of the three types of explanatory variables.

We used the “*randomForest*” package (Liaw and Wiener, 2002) for the R statistical language (R Core Team, 2013) to perform random forest calculations, sorting the importance of different factors based on the %IncMSE values obtained by the “*importance*” function in sequential order.

Finally, we also constructed partial regression plots to illustrate the effect direction (positive or negative) of each selected variable on wood density variation. Scatter plots were generated using the ‘*geom_point*’ function (Wickham, 2011), and a regression line was added to show the linear relationship using the ‘*geom_smooth*’ function.

## 3 Results

### 3.1 Patterns of wood density across biomes

**Fig. 2:**
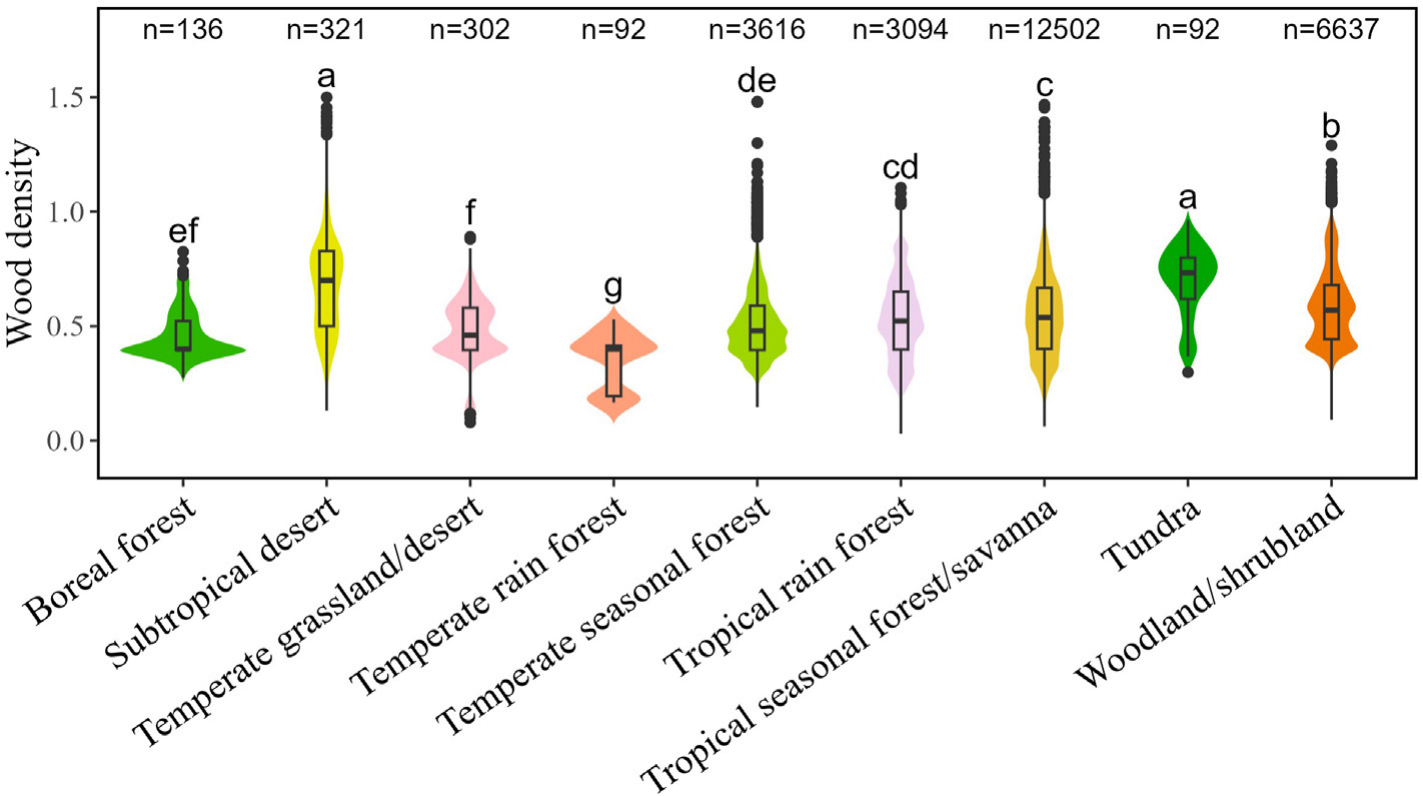
Patterns of wood density across different Whittaker's biomes. The black boxes within each violin plot show the median values and the lower and upper quartiles, the whiskers in each violin plot represent 1.5 interquartile range. Different lower-case letters adjoining the violin plots indicate the significant difference (*p* < 0.05) among different groups for wood density based on one-way ANOVA with the least significant difference post-hoc test. Bonferroni method was used to adjust p-values. The number above each violin plot is the number of records for the site–species combinations within that group.

Our results showed that wood density varied considerably across Whittaker's biomes, with the mean values being maximum in the subtropical desert and tundra, minimum in the temperate rainforest and boreal forest, and intermediate in other biomes (i.e., temperate grassland/desert, temperate seasonal forest, tropical rainforest, tropical seasonal forest/savanna and woodland/shrubland (Fig. 2 and Table S2).

### 3.2 Phylogenetic constraints in wood density in different plant groups

**Fig. 3:**
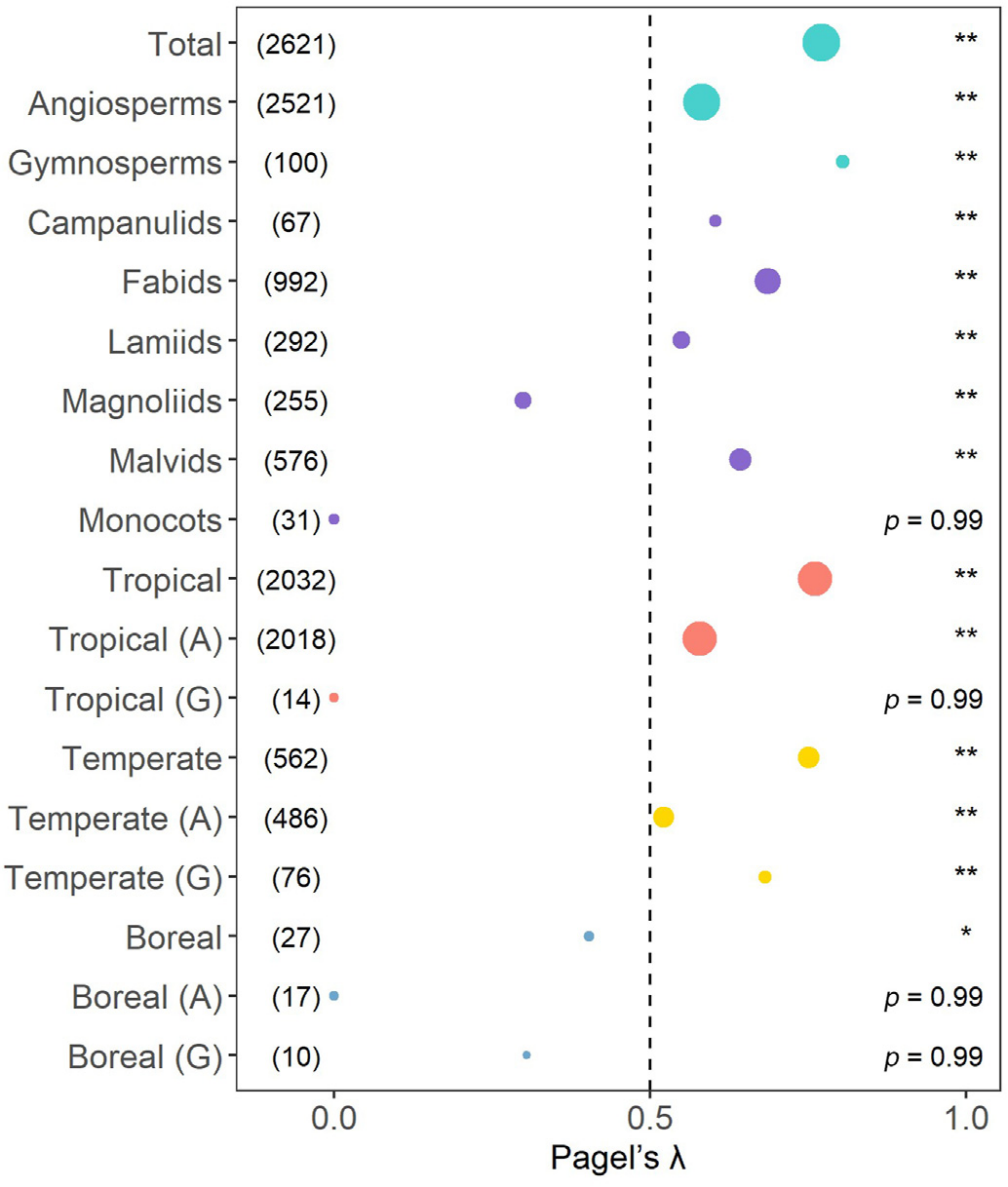
Results of phylogenetic signal for wood density in this study. The figure shows Pagel's λ statistics and associated *p* values for different groups of tree species. Total represents wood density dataset for 2621 species. Campanulids, Fabids, Lamiids, Magnoliids, Malvids and Monocots represent six main angiosperm subgroups in our dataset. Tropical, Temperate, and Boreal represent wood density data for tropical regions, temperate regions and boreal regions, respectively; the letters “A” and “G” represent angiosperms and gymnosperms, respectively. A number in parentheses represents the number of species. The size of each dot is proportional to the number of samples. Level of significance: ∗*p* < 0.05; ∗∗*p* < 0.01.

**Fig. 4:**
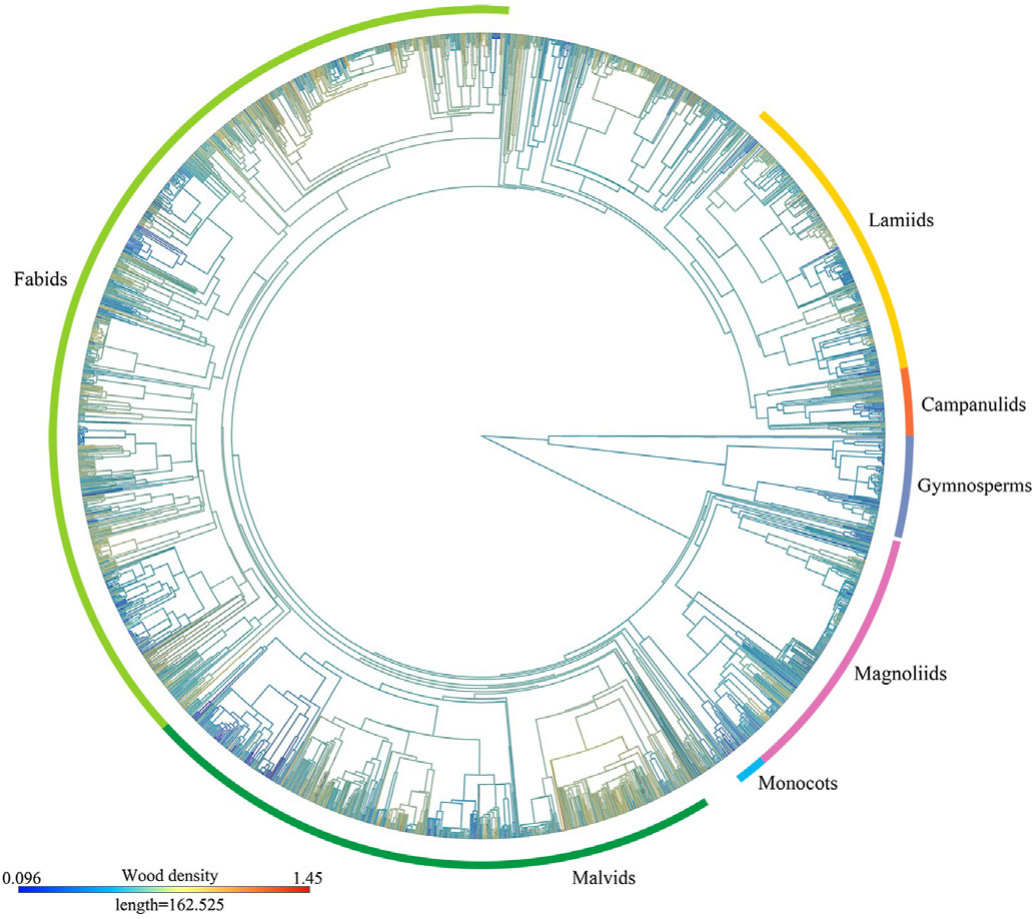
Phylogenetic tree of wood density for 2621 species investigated in this study. This was obtained using the ape package and the contMap function in R, assuming Brownian motion as a model for trait evolution, and then interpolated along the branches of the tree. The color gradient at the lower-left corner corresponds to the variation of color on the branches in the phylogenetic tree.

We analyzed the phylogenetic signals in wood density using Pagel's λ and found that tree species of both angiosperms and gymnosperms in different species groups had significant signals (λ > 0.50 with *p* < 0.01 in most cases; Fig. 3). For example, when all species (including both angiosperms and gymnosperms) were considered together, the global phylogenetic signal value for wood density was 0.77 (*p* < 0.01). We also found strong phylogenetic signals in both tropical species (λ = 0.76, *p* < 0.01) and temperate species (λ = 0.75, *p* < 0.01). However, tree species in the boreal zone had weaker phylogenetic signal of wood density (λ = 0.40, *p* < 0.01) than those in the other two climate zones. Our results show that closely related species have more similar wood densities than distantly related species (Fig. 4), indicating that evolutionary constraints play a key role in driving wood density variation.

The average of wood density values for angiosperms was larger than that for gymnosperms (0.57 ± 0.20 SD and 0.49 ± 0.11 SD, respectively; Fig. 1c). We conducted a comparison of phylogenetic constraints between angiosperms and gymnosperms and found some differences between the two groups (Fig. 3 and Table S3). The phylogenetic signal value within gymnosperms (λ = 0.81, *p* < 0.01) is larger than that within angiosperms (λ = 0.58, *p* < 0.01). In the tropics, wood density demonstrated significant phylogenetic signals for angiosperms (λ = 0.58, *p* < 0.01), but not for gymnosperms (λ < 0.01, *p* > 0.05). Conversely, gymnosperm trees (λ = 0.68, *p* < 0.01) showed greater phylogenetic constraints than angiosperm trees (λ = 0.52, *p* < 0.01) in temperate zones. Variation in wood density among climate zones was likely influenced by differences in tree form and wood structure between angiosperms and gymnosperms. In the boreal zone, where there are fewer species and extreme climatic conditions prevail, no significant phylogenetic signals were found when angiosperms and gymnosperms were analyzed separately (λ < 0.01 for angiosperms, λ = 0.31 for gymnosperms, *p* > 0.05 in both cases).

When the six major angiosperm lineages (i.e., Campanulids, Fabids, Lamiids, Magnoliids, Malvids and Monocots) were analyzed separately, significant phylogenetic signals were found in Campanulids (λ = 0.60, *p* < 0.01), Fabids (λ = 0.69, *p* < 0.01), Lamiids (λ = 0.55, *p* < 0.01) and Malvids (λ = 0.64, *p* < 0.01). Magnoliids showed low phylogenetic signals (λ = 0.30, *p* < 0.01), but no significant phylogenetic signal was found in Monocots (λ < 0.01, *p* > 0.05).

### 3.3 Relative contribution of environmental factors and evolutionary history to global wood density variation

**Fig. 5:**
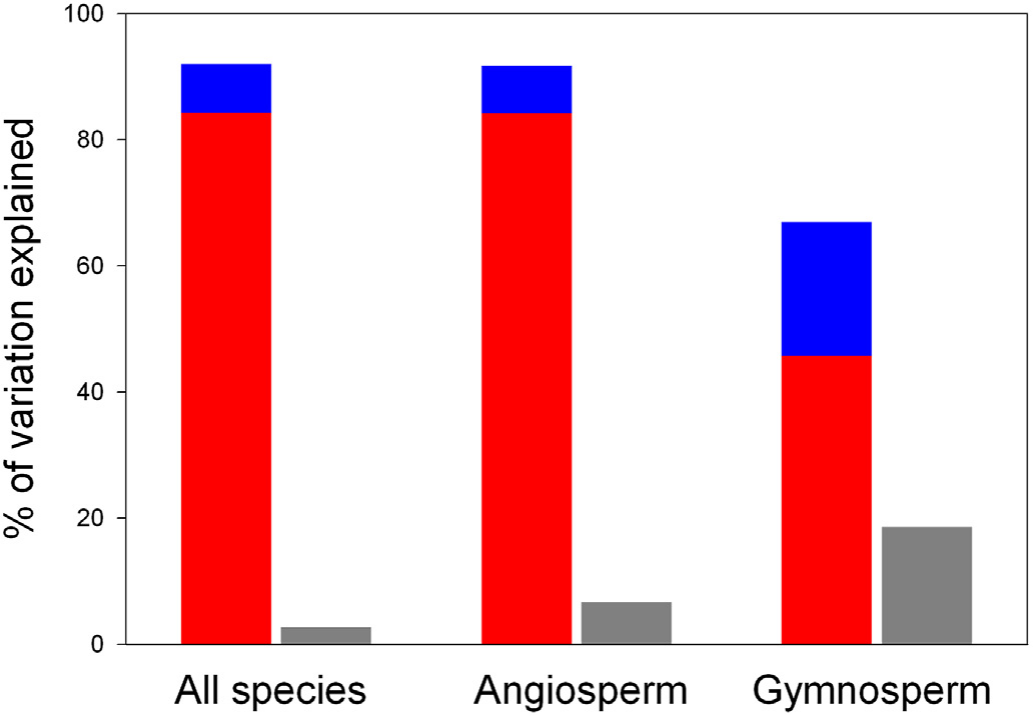
Variation in wood density explained by phylogeny (red), species (blue) and environmental variables (gray). Variations explained by phylogeny and species were estimated by Bayesian phylogenetic linear mixed models, which were reported in Table 1, and variation explained by environmental variables was estimated by multiple linear models, each of which included wood density as the dependent variable of the model and environmental variables as the independent variables of the model.

To investigate the relative importance of current environmental factors and evolutionary history on global wood density variation, we first identified important environmental factors based on model selection, which are MAT, MAP, clay and NDVI, and then used Bayesian phylogenetic linear mixed models to determine the variation in wood density that was explained by evolutionary history (represented by phylogeny and species), and used multiple linear regression models to determine the variation in wood density that was explained by environmental factors (we used the latter modeling approach in conjunction with the former modeling approach because the former modeling approach does not report the full proportion of the variation in the dependent variable explained by environmental variables). We found that evolutionary history outpaced environmental factors in explaining global variation in wood density. For example, for all species as a whole, current environmental factors explained only 2.7% of the variation in wood density variation, while phylogeny and species explained 84.3% and 7.7% of wood density variation, respectively (Table 1 and Fig. 5). For angiosperms, environmental factors explained 6.7% of the wood density variation, while phylogeny and species explained 84.2% and 7.5% of the wood density variation, respectively. For gymnosperms, environmental factors explained 18.5% of the wood density variation, while phylogeny and species explained 45.7% and 21.3% of the wood density variation, respectively (Table 1). When the variation in wood density was accounted for by phylogeny and species, environmental factors explained only 3% or less of the variation in wood density (i.e., values associated with R^2^m in Table 1). Thus, evolutionary history was far more important than the current environmental factors in explaining variation in global wood density.

**Table 1:** Results from Bayesian phylogenetic linear mixed model of wood density at site–species level, with fixed factors (i.e., environmental factors) and random factors (i.e., phylogeny + species) considered.

Variable	Post.mean	Lower 95% CI	Upper 95% CI	Eff.samp	*p*MCMC
**(1) All species**					
Intercept	−0.498	−1.661	0.760	2009	0.064
MAT	0.022	0.018	0.026	1700	<0.001
MAP	<−0.001	<−0.001	<−0.001	1700	<0.05
Clay	−0.001	−0.002	0.000	1769	<0.05
SOC	<−0.001	<−0.001	<−0.001	2271	<0.001
Model statistics: R^2^m = 0.007, R^2^c = 0.927, R^2^p = 0.843, R^2^s = 0.077					
**(2) Angiosperm**					
Intercept	−0.36	−1.226	0.59	1912	0.072
MAT	0.014	0.009	0.019	1498	<0.001
MAP	<−0.001	<−0.001	<−0.001	1700	<0.05
Clay	−0.002	−0.003	−0.001	1700	<0.001
NDVI	<−0.001	<−0.001	<−0.001	1700	<0.001
Model statistics: R^2^m = 0.003, R^2^c = 0.920, R^2^p = 0.842, R^2^s = 0.075					
**(3) Gymnosperm**					
Intercept	−1.819	−2.612	−0.896	1700	<0.001
MAT	0.049	0.039	0.059	1700	<0.001
MAP	<−0.001	<−0.001	<−0.001	1700	<0.01
Clay	0.002	< −0.001	0.004	1700	<0.05
NDVI	<−0.001	<−0.001	<−0.001	1700	0.063
Model statistics: R^2^m = 0.031, R^2^c = 0.702, R^2^p = 0.457, R^2^s = 0.213					

Note: The site–species level was analyzed by using the averaged wood density for each species within the same sampling site. Bayesian model: MAT + MAP + Clay + NDVI + (random = phylogeny + species). Abbreviations: MAT, mean annual temperature; MAP, mean annual precipitation; Clay, soil clay content; NDVI, Normalized Difference Vegetation Index; R^2^c, percentage of variance explained by all the model (fixed + random); R^2^m, percentage of the residual variance in wood density (i.e. the variance not explained by phylogeny and species) that was explained by fixed factors; R^2^p, percentage of variance explained by phylogeny; R^2^s, percentage of variance explained by species; Post.mean, posterior mean; Eff.samp, the effective sample size; *p*MCMC, *p*-value from Monte Carlo sampling by Markov Chain.

Our random forest (RF) model, which used phylogeny, species, MAT, MAP, clay and NDVI as six moderators, explained a significant proportion of global wood density variation; it also showed that phylogeny, species and MAP are the most important moderators (Figs. S3–S5).

## 4 Discussion

### 4.1 Significant differences in wood density among different biomes

We studied the global variation of wood density using an unprecedented and comprehensive dataset, and analyzing it by a Bayesian phylogenetic linear mixed model: we observed that at a global scale evolutionary history explained the most current variation (84.3%) of wood density across species (Table 1). We found higher wood density in subtropical desert relative to temperate rainforests is associated, to some degree, with lower MAP and NDVI (Table S2). Our findings on wood density distribution patterns differ from those of Yang et al. (2024) for some biomes. Future studies should reconcile discrepancies observed in different studies on wood density.

There was a discrepancy in the relationship between wood density and rainfall, with wood density found to be negatively regulated by MAP, contrary to some previous studies (Wiemann and Williamson, 2002; Zhang et al., 2011). Adequate water is key to tree growth and wood density, as density determines the variation in many characteristics related to the efficiency and integrity of water transport in the xylem and regulation of water balance, avoiding turgor loss (Hacke et al., 2001; Meinzer, 2003; Colgan et al., 2014; Kim et al., 2014). Scarcity of water appears to promote the growth of plants with high wood density on a global scale, consistent with the direct relationship between wood density and drought (Nabais et al., 2018; Pandey, 2021).

NDVI was also found to have a significant negative correlation with wood density, which reflects the diversity of ground vegetation and plant growth status (Pettorelli et al., 2011). When NDVI approaches to decrease, this would indicate low species richness and poor living conditions correlated with increased wood density. Further investigation into the correlation between NDVI data and the biological characteristics of trees, or the utilization of other biophysical parameters may contribute to enhancing the assessment of wood density variation based on current environment variation.

### 4.2 Phylogenetic constraint of wood density

The tendency of species to retain their ancestral traits, i.e., phylogenetic niche conservatism, is considered to strongly influence species distribution across environmental gradients. Species with shared evolutionary history tend to be adapted to similar environmental conditions since they inherit traits that determine their ecological niches from their ancestors (Wiens and Donoghue, 2004). A deep understanding of current environmental variables and evolutionary history underlying the large-scale variation in wood density helps us better understand the variations in wood density among geographic regions and across ecological gradients. However, characterizing the global variation of wood density has perpetually posed a challenge. In this study, we found strong phylogenetic signals of wood density, indicating that evolutionary history is a crucial factor in constraining the global distribution of wood density across tree species in both angiosperms and gymnosperms (Fig. 3 and Table S3). Using the λ statistics, we found significant phylogenetic signals (*p* < 0.05 for λ > 0.5) in all taxa, and observed phylogenetic signals for most plant groups except for Magnoliids, Monocots, tropical gymnosperm trees and boreal tree species, which each have relatively fewer species. The strong phylogenetic conservatism of wood density may be attributed to its fundamental functions, such as stem water transport, storage, and mechanical support (Pratt et al., 2007; Poorter et al., 2010). This is also related with the great number of genes that together codify for wood density (Li et al., 2012).

Our results showed that wood density is phylogenetically conserved in tropical and temperate zones, but not in cold zones, which is consistent with previous studies (Baker et al., 2004; Cavender-Bares et al., 2004; Chave et al., 2006; Swenson and Enquist, 2007). For example, Swenson and Enquist (2007) found strong phylogenetic conservation in wood density of tropical and temperate tree species. When angiosperms and gymnosperms were analyzed separately, it was suggested that there are similarities and differences in phylogenetic constraints of the two groups across different climatic zones (Figs. 4, S1 and S2). In tropical communities, phylogenetic constraints affected angiosperms but not gymnosperms. This could be because angiosperms have higher hydraulic conductivity, allowing them to outcompete gymnosperms in less hydraulically stressful environments and lower latitudes (Bond, 1989). Angiosperm trees also have, in general, higher wood density than gymnosperm trees (Fig. 1c; also see MacFarlane, 2020), which provides them with stronger mechanical support and a competitive edge (Swenson and Enquist, 2007). Therefore, in resource-rich tropical forests, phylogeny plays a significant role in the evolutionary selection of angiosperms. However, the low number of gymnosperm species together to the possibility of a convergent and/or divergent fast evolution of wood density in the last millions of years among this set of species can have diminished the phylogenetic signal but maintained or increased the species differences not directly related to long-term evolutive process. Our results show that at a global scale, the legacy effects not due to phylogeny explained 7.5% and 21.3% of the total variation in angiosperms and gymnosperms, respectively (see Table 1); in contrast, those due to phylogeny explained 84.2% and 45.7% of the total variation in the two groups, respectively (Table 1). Thus, both groups have high evolutionary legacy effects in explaining current variation in wood density, but in angiosperms this is mainly due to evolutionary process whereas in gymnosperms a great part is not related with phylogeny (Table 1). Gymnosperms appeared much earlier in the evolutionary history of our planet, compared to angiosperms, but they were, in most places, replaced by the most modern angiosperms. Accordingly, the most gymnosperm species have been more recently “readapted” in general to more harsh environments with higher levels of perturbations or, in most cases, to poorer resource availability and, thus, stressful conditions (Brodribb et al., 2012; Choat et al., 2012; Johnson et al., 2012). Thus, gymnosperms as a group of plants have probably been subjected to more intense phenomena of recent convergent and divergent evolution diminishing the signal of long-time evolution (phylogenetic signal) in explaining wood density variation among species. Therefore, gymnosperms could compete with success *in situ* whereas angiosperms are unable to realize their maximum growth potential because of environmental limitations on photosynthesis and growth (Bond, 1989; Brodribb et al., 2012).

Consistent with this general trend, in temperate communities, the phylogenetic signal of wood density in gymnosperms was higher than that in angiosperms (Fig. 3). Compared with tropical communities, greater wood density variation was observed in gymnosperms than in angiosperms. Gymnosperm trees have a more efficient wood construction cost to xylem safety trade-off than angiosperm trees, which contributes to greater survival and higher levels of variation (Swenson and Enquist, 2007). In contrast, hydraulically stressful environments limit evolutionary variation in angiosperm wood characteristics (Weiher and Keddy, 1995).

In the boreal zone, no significant evolutionary constraints were found on wood density for either angiosperms or gymnosperms. This may be, at least in part, because of relatively harsh climate in the boreal zone, few tree species in either group occur in this climatic zone, and these few species belong to few, closely related clades in a relatively narrow phylogenetic scope; as a result, phylogenetic signal in wood density for trees in the boreal zone would presumably be weak, as observed in this study. Furthermore, trees in both angiosperms and gymnosperms have adapted to the harsh environment and evolved convergent characteristics due to extreme environmental conditions such as severe lack of light and long periods of cold, triggering a convergence evolution with a driving evolutive force by the need to adapt to harsh cold conditions for all species. The non-tolerant and non-resistant morphological characteristics of angiosperms and gymnosperms were filtered by habitat factors and evolved to produce similar growth patterns (Cadotte and Tucker, 2017). Therefore, they tend to have more conservative strategies and lower levels of plasticity (Grime, 1977; Güsewell, 2004). As found in several previous studies, species planted in the same habitat as a common garden tend to develop convergent traits. This is precisely the case in our study of the boreal zone, where more stress-tolerant species commonly show narrower variation in wood density (Güsewell et al., 2005; Yu et al., 2011; Peñuelas et al., 2019).

### 4.3 Evolutionary history explains a great amount of global wood density variation

We investigated the relative importance of environmental factors and evolutionary history in explaining global wood density variation and found that evolutionary history (represented by both phylogeny and species) explained a much larger proportion of wood density variation than current environmental factors (Table 1 and Figs. 5, S3−S5). Yang et al. (2024) reported that climatic predictors, vegetation characteristics, and edaphic properties explain, respectively, 49–63%, 25–31%, and 11–16% of spatial variation in wood density worldwide. However, these percentages reflect the relative importance of each group (vegetation, climatic, and edaphic) to the total of the actual explained variation, and the sum of the relative importance is 100% (as shown in fig. 4 of their study). In addition, their study did not considered the effect of evolutionary history on wood density. Thus, their results are not comparable with ours. Our study indicates that considering evolutionary history is important in future studies on wood density.

Our finding shows that the combination of phylogeny and species explained much more variation in a biological trait (i.e. wood density) than current environmental variables is, to a large degree, consistent to the findings of previous studies on other biological traits. For example, Sardans et al. (2021) found that phylogeny and species explained, on average, 80.9% and 5.58% of the variation of foliar elemental composition, respectively; after the variation in foliar elemental composition was accounted for by phylogeny and species, the climatic variables and N deposition examined in their study only explained, on average, 2.26% of the variation in the residual of foliar elemental composition (note that Sardans et al. (2021) interpreted this part of the explained variation as the variation explained by the climatic variables and N deposition).

Phylogeny reflects the long-term evolutionary history, through which species adapt to abiotic and biotic stressors caused by climate, soil, and other species (Moritz and Faith, 2002), including information on adaptation and differentiation of different clades at both deep and shallow evolutionary history, whereas species is linked to more recent evolutionary processes, including more genetic and epigenetic factors (Sardans et al., 2021; Vallicrosa et al., 2022; Huang et al., 2023a). It is suggested that distantly related lineages may have been exposed to similar environmental conditions, leading to parallel selection that determines similar morphological and functional characteristics in species of different lineages (Sardans et al., 2021). On the other hand and mainly in resource-rich environments with higher niche differentiation due to the strong competition pressure to compete and uptake the existing resources (e.g., water, nutrients, light), species of similar clades can have suffered a fast niche differentiation and divergent evolution pressure to avoid direct competition (Sardans et al., 2021). This implies that distantly related lineages may converge evolutionarily under similar environmental conditions, resulting in similar wood density in different clades (Smith and Donoghue, 2010; Nyari and Reddy, 2013), but also that species coexisting in the same community can diverge to avoid direct competition (Pinsky, 2019; Sardans et al., 2021). For instance, a warmer climate can accelerate the evolution of various traits differently in different lineages (Bellard et al., 2012).

Moreover, it is logical that when evolutionary variables such as phylogeny and species (legacy effects) are included as random factors in a model, a part of the variability explained by current environmental circumstances are also explained by legacy effects. For instance, several tropical species that have acquired their traits in recent millions of years could live in environmental circumstances relatively similar to the current ones, and this can be also applied to several other species that along their evolutionary process have lived for a long period in climate and environmental circumstances that are similar to the present environmental circumstances. Thus, when including legacy effects in the model, it explains a part of the variability due to current environmental variables, and thus the current environmental variables included in the model explained a smaller part of the variance than that when legacy effects are not included in the model.

Considering that both evolutionary history information and current environmental factors jointly regulate large-scale variation in plant functional traits, including wood density, our results further suggest that the variation of a biological trait associated with species and phylogeny must be credited, in addition to the site-associated current environmental factors, when estimating and projecting the global wood density variation.

### 4.4 Limitations

There are some limitations in our study. First, most of the studied sites were from Europe, Asia and South America, with few data from Africa. Second, our study included only 4.3% of the *ca*. 61,000 tree species worldwide (Qian et al., 2019). Third, stand structure factors, which are difficult to collect, such as stand density, canopy density and stand age (Lee et al., 2004), were not included in the analysis. While NDVI was significantly correlated with these biophysical parameters and has the potential to explain the variation of woody biomass (Boelman et al., 2003; Cabrera-Bosquet et al., 2011; Wani et al., 2021), biological data such as stand density, crown density and stand age would help better explain the influence of those factors on wood density. Finally, the random forest models used in the present study did not fully account for the variation in wood density, and more variables, including remote sensing parameters (e.g., GEDI data), may be needed to better understand the main drivers of wood density. Future studies may include these and other variables, when data for them are available.

## 5 Conclusion

Our study utilized a comprehensive global dataset to show that phylogeny plays a significant role in shaping wood density patterns. This study revealed that (1) wood density exhibited a significant and relatively strong phylogenetic signal; (2) evolutionary history, consisting of both phylogeny and species, largely outperformed present-day environmental conditions in explaining global wood density variation, and (3) wood density showed significant biogeographical patterns at the global scale and varied remarkably across different biomes. These results collectively suggest that prioritizing evolutionary history in future wood density investigations will enhance our knowledge of forest wood and carbon variations. Moreover, this study sheds light on the role of evolutionary legacy in shaping current variation of at least some functional traits, as observed on wood density in this study and observed on foliar nitrogen and phosphorus concentrations in previous studies.

## Data accessibility statement

Data used in this study were included in the manuscript or supplementary information.

## CRediT authorship contribution statement

**Fangbing Li:** Writing – original draft, Data curation, Conceptualization. **Hong Qian:** Writing – review & editing, Writing – original draft, Formal analysis, Data curation. **Jordi Sardans:** Writing – review & editing. **Dzhamal Y. Amishev:** Writing – original draft. **Zixuan Wang:** Writing – original draft. **Changyue Zhang:** Writing – original draft, Data curation. **Tonggui Wu:** Writing – original draft. **Xiaoniu Xu:** Writing – original draft. **Xiao Tao:** Writing – original draft. **Xingzhao Huang:** Writing – review & editing, Writing – original draft, Investigation, Formal analysis, Data curation, Conceptualization.

## Declaration of competing interest

The authors have no competing interest to declare.
