# Supplementary material for: Evolutionary history shapes variation of wood density of tree species across the world
Source: Plant Divers. 2024 Apr 10;46(3):283–93. doi: 10.1016/j.pld.2024.04.002 (PMC11119544; doi:10.1016/j.pld.2024.04.002)
Supplement: Multimedia component 2 [file mmc2.doc]

**Supporting Information for**

**Evolutionary history shapes variation of wood density of tree species across the world**

**Table S1** Site characteristics of the wood density in compiled dataset.

| Parameter | Unit | Natural terrestrial ecosystems | |
| --- | --- | --- | --- |
|  |  | Range | N |
| Latitude | ° | -46.07-70.83 | 27297 |
| Longitude | ° | -155.48-176.22 | 27297 |
| Family |  |  | 27297 |
| Genus |  |  | 27297 |
| Species |  |  | 27297 |
| Mean annual temperature | °C | -13.40-28.10 | 27297 |
| Mean annual precipitation | mm yr^-1^ | 4-6248 | 27297 |
| Site aspect | ° | 1-255 | 27297 |
| Site slope | ° | 0.03-31.78 | 27297 |
| Soil clay content | % | 1-74 | 27297 |
| Soil organic carbon | mg g^-1^ | 1.90-352.70 | 27297 |
| Normalized Difference Vegetation Index |  | 502-9671 | 27297 |

**Table S2** **Summary of the statistical metrics of wood density and the examined seven environmental factors with different Whittaker’s biomes.** Mean and standard error (SE) are shown. The important environmental factors include mean annual temperature (MAT); mean annual precipitation (MAP); soil clay content (Clay); site slope (Slope); site aspect (Aspect); soil organic carbon (SOC); NDVI, Normalized Difference Vegetation Index, respectively. Different lower-case letters adjoining the mean values indicate significant difference (p<0.05) for the wood density based on one-way analysis of variance and least significant difference post-hoc test.

| Biome group | Wood density (g cm^-3^) | Mean annual temperature (°C) | Mean annual precipitation (mm yr-1) | Site aspect (°) | Site slope (°) | Soil clay content (%) | Soil organic carbon (mg g^-1^) | Normalized Difference Vegetation Index |
| --- | --- | --- | --- | --- | --- | --- | --- | --- |
| Boreal forest | 0.46±0.11^f^ | 1.22±1.25^f^ | 696±201^e^ | 171±70^bc^ | 4.89±6.11^b^ | 14±10^g^ | 18.83±1.77^bcd^ | 7587±1616^ab^ |
| Subtropical desert | 0.70±0.25^a^ | 23.01±2.93^b^ | 294±207^f^ | 146±87^d^ | 1.00±1.55^e^ | 28±18^cd^ | 7.66±3.58^e^ | 2128±1112^g^ |
| Temperate grassland/desert | 0.47±0.15^f^ | 11.61±4.87^d^ | 333±117^f^ | 170±82^c^ | 2.02±2.33^d^ | 27±11^de^ | 10.06±4.05^de^ | 4149±1391^f^ |
| Temperate rainforest | 0.35±0.12^g^ | 14.97±3.73^c^ | 2387±241^b^ | 165±90^c^ | 7.04±6.16^a^ | 23±6^f^ | 15.73±5.76^cde^ | 6906±989^d^ |
| Temperate seasonal forest | 0.51±0.16^e^ | 10.62±5.18^e^ | 1197±372^d^ | 170±83^c^ | 6.39±6.08^a^ | 29±17^c^ | 25.60±0.40^b^ | 7291±1001^c^ |
| Tropical rainforest | 0.53±0.17^d^ | 25.48±0.89^a^ | 3122±435^a^ | 139±73^d^ | 2.44±2.63^d^ | 31±15^b^ | 24.01±3.12b^c^ | 7711±1442^a^ |
| Tropical seasonal forest/savanna | 0.55±0.19^c^ | 22.98±2.69^b^ | 1561±314^c^ | 204±73^a^ | 4.09±3.94^c^ | 33±13^a^ | 28.56±6.46^a^ | 7421±1165^b^ |
| Tundra | 0.69±0.16^a^ | -9.80±1.81^g^ | 200±82^g^ | 186±24^b^ | 6.47±7.12^a^ | 25±7^df^ | 18.63±4.58^bcd^ | 6162±639^e^ |
| Woodland/shrubland | 0.59±0.17^b^ | 11.72±3.54^d^ | 693±169^e^ | 164±79^c^ | 4.58±4.27^b^ | 26±13^df^ | 13.05±0.29^de^ | 6122±1405^e^ |

**Table S3** Phylogenetic signal of wood density for different groups of tree species. Abbreviations in the first column: A = angiosperm, and G = gymnosperm.

| Plant group | records | No. of species | Pagel's λ | p-value |
| --- | --- | --- | --- | --- |
| All species | 27297 | 2621 | 0.771 | <0.01 |
| Angiosperms | 19788 | 2521 | 0.582 | <0.01 |
| Gymnosperms | 7509 | 100 | 0.805 | <0.01 |
| Campanulids | 378 | 67 | 0.603 | <0.01 |
| Fabids | 9103 | 992 | 0.686 | <0.01 |
| Lamiids | 2007 | 292 | 0.550 | <0.01 |
| Magnoliids | 1390 | 255 | 0.299 | <0.01 |
| Malvids | 4623 | 576 | 0.643 | <0.01 |
| Monocots | 83 | 31 | <0.001 | 0.99 |
| Tropical | 12272 | 2032 | 0.760 | <0.01 |
| Tropical (A) | 12213 | 2018 | 0.578 | <0.01 |
| Tropical (G) | 59 | 14 | <0.001 | 0.99 |
| Temperate | 14567 | 562 | 0.752 | <0.01 |
| Temperate (A) | 7318 | 486 | 0.522 | <0.01 |
| Temperate (G) | 7249 | 76 | 0.681 | <0.01 |
| Boreal | 458 | 27 | 0.403 | <0.05 |
| Boreal (A) | 257 | 17 | <0.001 | 0.99 |
| Boreal (G) | 201 | 10 | 0.305 | 0.99 |


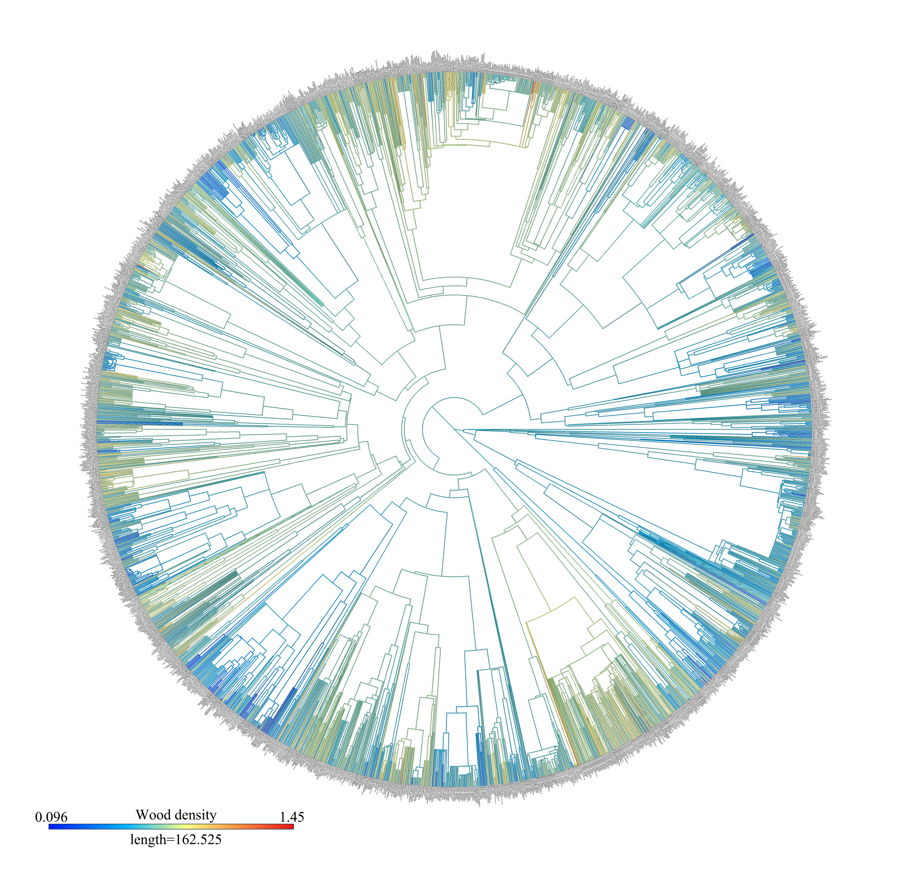


**Fig. S1** Phylogenetic tree of wood density for angiosperms (2,521 species) in this study. This was obtained using the ape package and the contMap function in R, assuming Brownian motion as a model for trait evolution, and then interpolated along the branches of the tree.


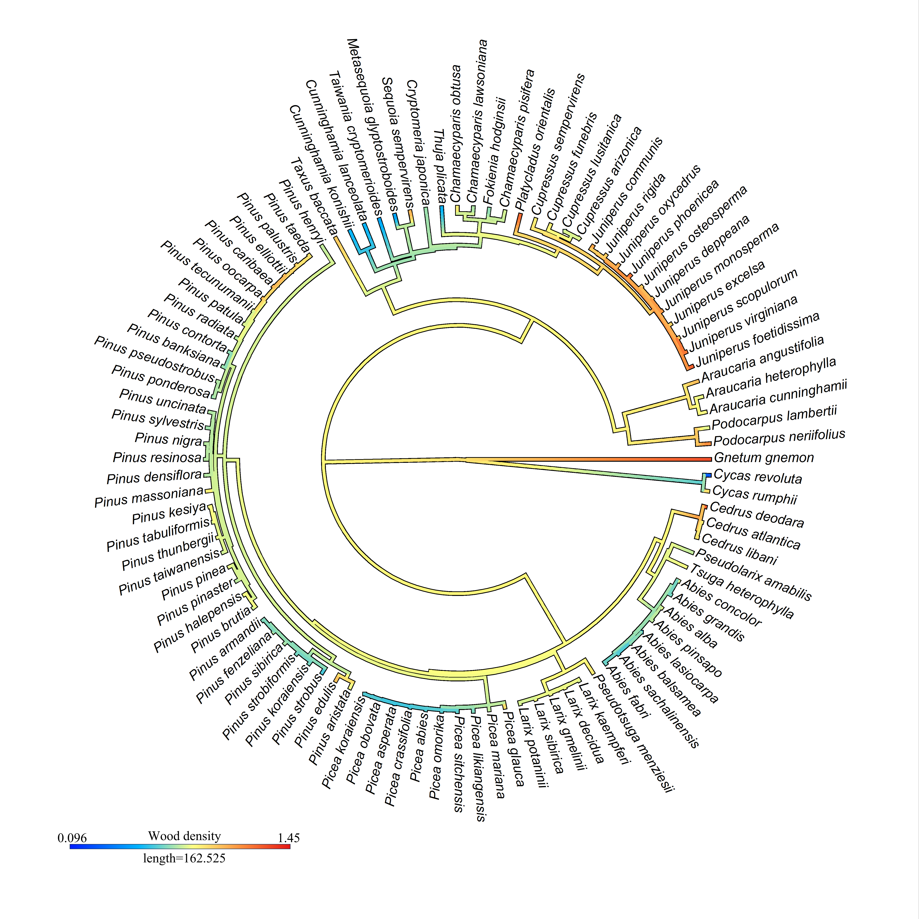


**Fig. S2** Phylogenetic tree of wood density for gymnosperms (100 species) in this study. This was obtained using the ape package and the contMap function in R, assuming Brownian motion as a model for trait evolution, and then interpolated along the branches of the tree.


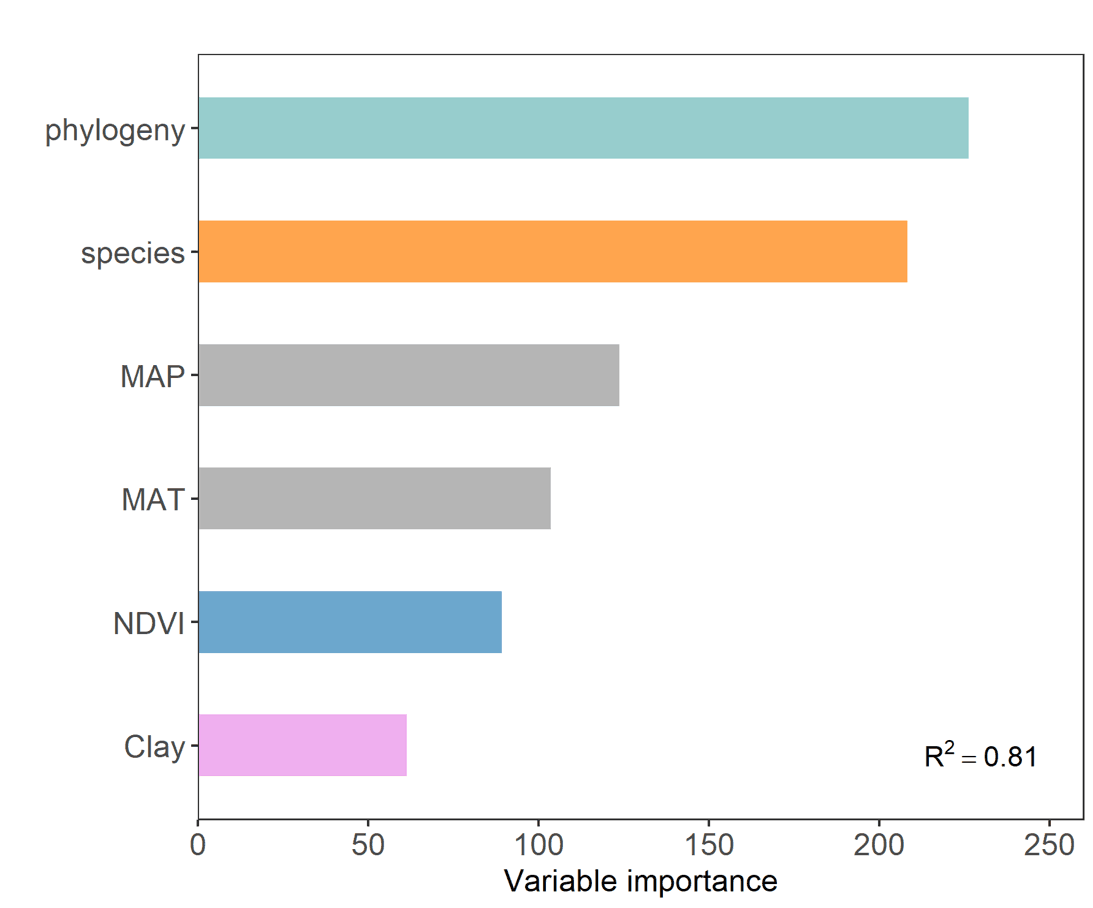


**Fig. S3 Relative importance of explanatory variables for predicting wood density using a random forest analysis.** R^2^ value indicates the total proportion of variation explained by the random forest model. Moderators include phylogeny, species, mean annual temperature (MAT), mean annual precipitation (MAP), Normalized Difference Vegetation Index (NDVI) and soil clay content (Clay).


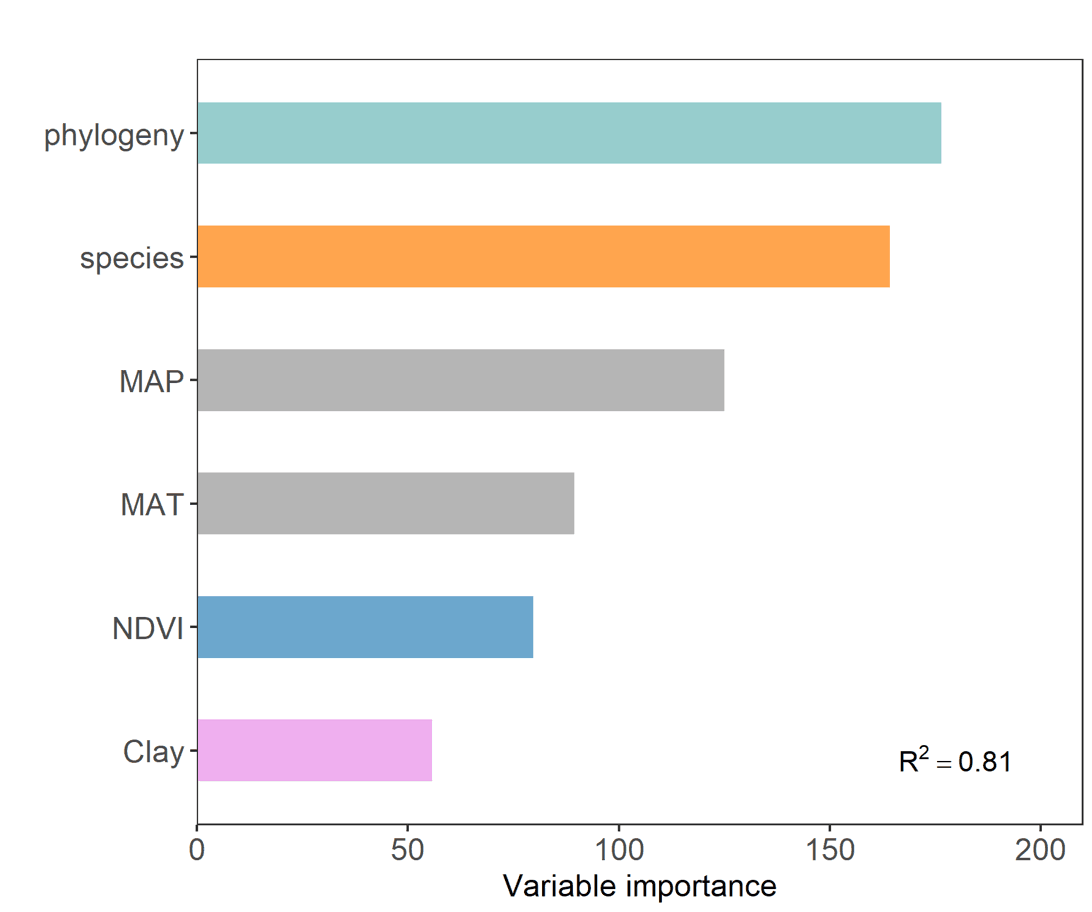


**Fig. S4 Relative importance of explanatory variables for predicting angiosperm wood density using a random forest analysis.** R^2^ value indicates the total proportion of variation explained by the random forest model. Moderators include phylogeny, species, mean annual temperature (MAT), mean annual precipitation (MAP), Normalized Difference Vegetation Index (NDVI) and soil clay content (Clay).


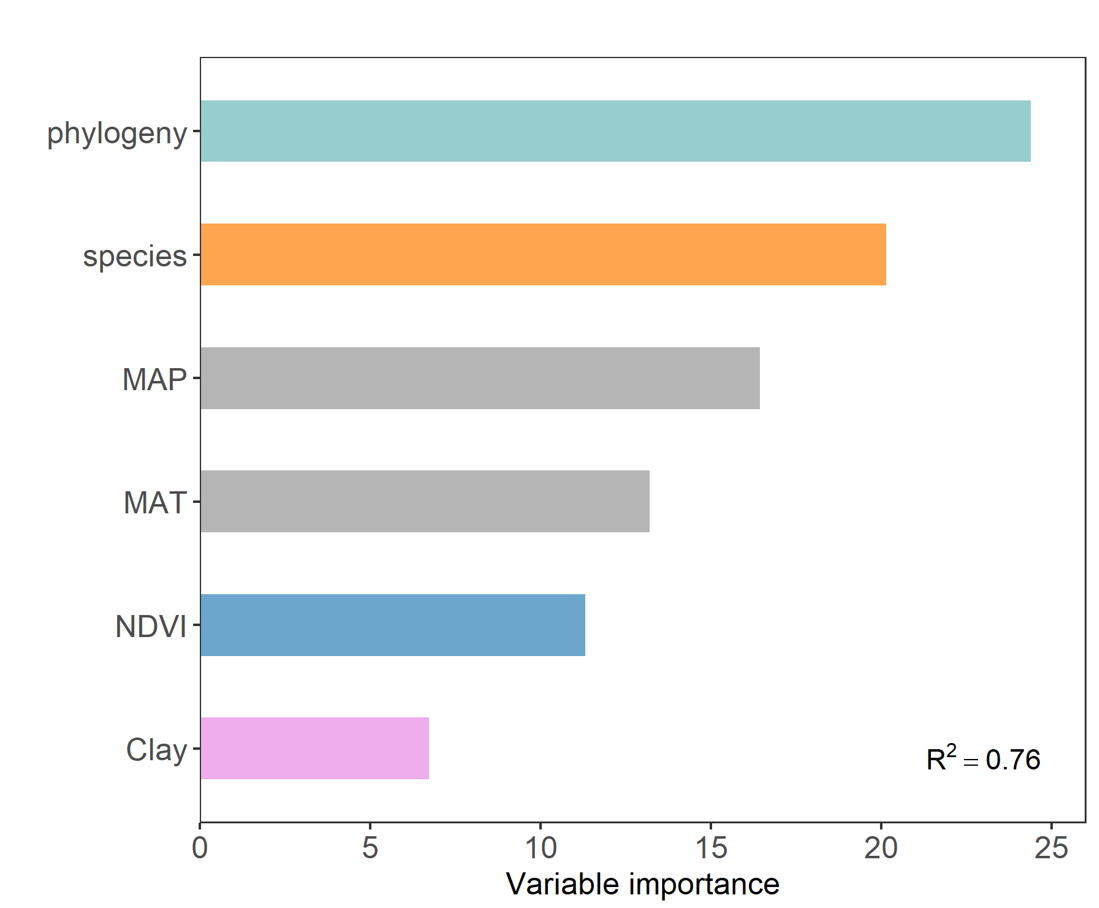


**Fig. S5 Relative importance of explanatory variables for predicting gymnosperm wood density using a random forest analysis.** R^2^ value indicates the total proportion of variation explained by the random forest model. Moderators include phylogeny, species, mean annual temperature (MAT), mean annual precipitation (MAP), Normalized Difference Vegetation Index (NDVI) and soil clay content (Clay).
